# Proliferation rates and gene expression profiles in human lymphoblastoid cell lines from patients with depression characterized in response to antidepressant drug therapy

**DOI:** 10.1038/tp.2016.185

**Published:** 2016-11-15

**Authors:** J Breitfeld, C Scholl, M Steffens, K Brandenburg, K Probst-Schendzielorz, O Efimkina, D Gurwitz, M Ising, F Holsboer, S Lucae, J C Stingl

**Affiliations:** 1 Research Division, Federal Institute for Drugs and Medical Devices (BfArM), Bonn, Germany; 2 Institute of Clinical Pharmacology and Pharmacology of Natural Products, University of Ulm, Ulm, Germany; 3 Faculty of Medicine, Tel Aviv University, Tel Aviv, Israel; 4 Max Planck Institute of Psychiatry, Munich, Germany; 5 HMNC Holding GmbH, Munich, Germany; 6 Center for Translational Medicine, Bonn University Medical School, Bonn, Germany

## Abstract

The current therapy success of depressive disorders remains in need of improvement due to low response rates and a delay in symptomatic improvement. Reliable functional biomarkers would be necessary to predict the individual treatment outcome. On the basis of the neurotrophic hypothesis of antidepressant's action, effects of antidepressant drugs on proliferation may serve as tentative individual markers for treatment efficacy. We studied individual differences in antidepressant drug effects on cell proliferation and gene expression in lymphoblastoid cell lines (LCLs) derived from patients treated for depression with documented clinical treatment outcome. Cell proliferation was characterized by EdU (5-ethynyl-2'-deoxyuridine) incorporation assays following a 3-week incubation with therapeutic concentrations of fluoxetine. Genome-wide expression profiling was conducted by microarrays, and candidate genes such as betacellulin—a gene involved in neuronal stem cell regeneration—were validated by quantitative real-time PCR. *Ex vivo* assessment of proliferation revealed large differences in fluoxetine-induced proliferation inhibition between donor LCLs, but no association with clinical response was observed. Genome-wide expression analyses followed by pathway and gene ontology analyses identified genes with different expression before vs after 21-day incubation with fluoxetine. Significant correlations between proliferation and gene expression of *WNT2B*, *FZD7*, *TCF7L2*, *SULT4A1* and *ABCB1* (all involved in neurogenesis or brain protection) were also found. Basal gene expression of *SULT4A1* (*P*=0.029), and gene expression fold changes of *WNT2B* by *ex vivo* fluoxetine (*P*=0.025) correlated with clinical response and clinical remission, respectively. Thus, we identified potential gene expression biomarkers eventually being useful as baseline predictors or as longitudinal targets in antidepressant therapy.

## Introduction

The therapy of depression is characterized by response rates around 60% and difficulties in the early evaluation of individual therapy success owing to delayed clinical improvement that may take from weeks up to several months.^1, 2^ So far, it is not yet possible to predict the individual treatment outcome of depressive patients owing to a lack of predictive biomarkers. According to the neuroplasticity hypothesis of the antidepressants' action, which is based on both animal and human cell models, antidepressants act—at least in part—by increasing proliferation of neuronal stem cells.^3, 4^ Furthermore, depressed patients have been reported with volume reductions in hippocampus and other brain regions,^5^ which has been observed to be reversed after successful antidepressant therapy, apparently owing to antidepressant-induced triggering of neural plasticity.^6^ As cerebral remodeling processes are complex and take many weeks, this explains the observed delay in symptomatic improvement.^7^ Consequently, the late-onset action of antidepressant drugs in the treatment of depression is hypothesized owing to changes in neuroplasticity resulting from these proliferative effects in the hippocampus.^8^

Individual differences in antidepressant-mediated modulation of cell growth were observed in human blood-derived lymphoblastoid cell lines (LCLs): sensitivity to paroxetine was measured by effects on *ex vivo* cell proliferation to identify potential gene and miRNA antidepressant response biomarkers.^9, 10^ Assuming that inter-individual variations in antidepressant effects on cell proliferation rates may serve as surrogate indicators for individual treatment efficacy,^8, 11, 12^ we used LCLs from depressed patients to study the effect of fluoxetine on cellular proliferation rates and their association with clinical response data. Furthermore, genome-wide gene expression analyses may further be used to identify tentative cell proliferation-associated biomarkers. Here we applied phenotypic screening of antidepressant effects on cell proliferation, combined with genome-wide expression profiling, for identifying tentative antidepressant response biomarkers that may assist in the early identification of treatment-resistant depression patients.

## Materials and methods

### Patients and cell lines

Epstein–Barr virus-transformed LCLs were generated in a subset of patients from the Munich Antidepressant Response Signature (MARS) project. The MARS study is a naturalistic clinical study on antidepressant drug response designed for pharmacogenetics analyses of antidepressant drug response biomarkers as described earlier.^2, 13, 14^ From the available LCLs from patients, various cell lines were picked for experiments (*n*=10 for microarray analysis, and *n*=25 responder and *n*=25 non-responder to antidepressant drug treatment for proliferation phenotyping). For cell line selection, the response and non-response statuses were considered after 8 weeks of antidepressant drug treatment (study population parameters are summarized in Table 1; drug response profiles are listed in Supplementary Figure 1 and Supplementary Table 2). LCLs were gained by Epstein–Barr virus transformation from full EDTA (ethylenediaminetetraacetic acid)-blood samples provided by the MARS patients admitted to the hospital of the Max Planck Institute of Psychiatry in Munich, Germany, for depression treatment.^2^ The study was approved by the Ethical Committee of the Medical Faculty at the Ludwig-Maximilian University. The participating patients gave verbal and written informed consent to provide biomaterial for the study of antidepressant response biomarkers also including transformation of blood lymphocytes into cell lines. MARS is an observational study of depressed patients being treated according to the attending physician's choice. Depressive symptoms were rated by the 21-item HDRS (Hamilton Depression Rating Scale) at weeks 0, 5 and 8 after study inclusion.^15^ Response was defined as HDRS reduction of at least 50% (compared with initial values at study inclusion) and remission was defined as a total reduction of HDRS to values smaller than 8.^16^

### Generation and cultivation of lymphoblastoid cell lines

LCLs were generated from lymphocytes isolated from blood samples through Epstein–Barr virus transformation.^17, 18^ Peripheral blood mononuclear cells were isolated by density gradient centrifugation using Ficoll, resuspended in Epstein–Barr virus supernatant from B95-8 cell line, and 100 μl were seeded into wells of a 48-well cell culture plate. After the addition of 200 μl Roswell Park Memorial Institute (RPMI) medium (containing 20% fetal calf serum (FCS)) per well, the cells were incubated at 37 °C in a humidified CO_2_ incubator (with 5% CO_2_) for 5 days. Subsequently, one volume of fresh RPMI medium (containing 20% FCS) and cyclosporine A (Sigma-Aldrich, St Louis, MO, USA) in a final concentration of 1 μg ml^−1^ were added. On day 25 after isolation, the cells from different wells of the same sample were pooled and further cultivated with exchange of the medium (containing 15% FCS) every second day. The cell identity was tested using the T- and B-cell specific antibodies CD3, CD19 and CD45 (BD Tritest Kit, Becton Dickinson, Heidelberg, Germany) through flow cytometry. The cells were cryo-stored in 90% FCS and 10% dimethyl sulfoxide. The LCLs were cultured in RPMI medium supplemented with 15% FCS, antibiotics (100 μg ml^−1^ penicillin, 100 μg ml^−1^ streptomycin) and a final concentration of 4 mm l-glutamine. The cells were incubated at 37 °C in a humidified CO_2_ incubator (with 5% CO_2_) in cell culture flasks.

### Incubation with antidepressants and LCL cell proliferation assay

EdU incorporation assays (Life Technologies, Carlsbad, CA, USA) were carried out according to the manufacturer instructions in technical and biological duplicates. Fluoxetine was chosen as antidepressant drug because its proliferative features are well studied,^19, 20, 21^ it showed the most distinct effects in preceding experiments using LCLs, and most of the MARS patients under antidepressant monotherapy received selective serotonin reuptake inhibitor antidepressant drugs. Mock-treated control cultures were grown in parallel, and cell density was set to 3 × 10^5^ cells per milliliter every second day. The incubation periods and fluoxetine effects on cell proliferation were tested at 7, 14 and 21 days of incubation, and it turned out that largest effects were observed after 21 days of continuous incubation with fluoxetine at a concentration of 0.5 μg ml^−1^ (including change of fresh medium every second day). The period of 21 days incubation with fluoxetine was therefore chosen for *ex vivo* phenotyping of the entire LCL panel. Fluoxetine was purchased from Sigma-Aldrich and stock solutions were prepared in dimethyl sulfoxide.

### Nucleic acid extraction

Nucleic acid extraction was performed with the AllPrep DNA/RNA Mini Kit (Qiagen, Hilden, Germany). Nucleic acid concentrations were quantified using a NanoDrop Spectrophotometer (Thermo Scientific, Darmstadt, Germany).

### Whole genome expression profiling

Microarray analyses were performed in 10 cell lines (untreated, and after 21 days of incubation with 0.5 μg ml^−1^ fluoxetine) using Agilent One Color Microarray Technology (Waldbronn, Germany; SurePrint G3 Human Gene Expression 8 × 60K Microarray Kit) containing probes for >27 000 transcripts. RNA quality was determined with Agilent 2100 Bioanalyzer and a total of 100 ng RNA was used for reverse transcription and labeling. The generation of complementary DNA (cDNA) was conducted with T7 promoter primers in a total reaction volume of 10 μl (containing 0.1 μm DTT, 5 μm dNTP mix and 1.2 μl RNase inhibitor in first-strand buffer) incubated for 2 h at 40 °C followed by 15 min at 70 °C. The labeling was performed for 2 h at 40 °C after the addition of NTP mix, T7 RNA polymerase and cyanin 3-CTP. After column-based purification of labeled complementary RNA, hybridization was carried out for 17 h and fluorescence intensities were measured by SureScan Microarray Scanner (Agilent). Data were normalized and summarized with the multiaverage method. Data analysis was conducted using GeneSpring (Agilent) and initially, the probeset was filtered on data files (control type 0) with the condition that at least 100% of the values in any one condition are within the expected range.

The differential gene expression was rated in pairs with fold-change cutoff of 2 and significance value of *P<*0.05 (uncorrected). Pathway analysis (single-experiment analysis) was performed using the imported pathway database from GenMAPP Pathway Markup Language and an uncorrected *P*-value cutoff of *P*<0.05 and focused on pathways inversely regulated in responder and non-responder indicator cell lines. Indicator cell lines are characterized by a rectified clinical response status, proliferation status and hierarchical clustering status: cell lines 24DC and 275U served as positive indicator cell lines and derived from clinical responders, they were *in vitro* proliferators and showed strong gene expression changes after treatment with fluoxetine, whereas cell line 278H was used as a negative indicator cell line. Gene ontology analysis was carried out using the web-based STRING database.^22^ Systematic search of central nervous system annotations were carried out using the gene names and one of the following terms: brain, neuron, neurogenesis, neural plasticity, proliferation, depression or antidepressant. Microarray data were deposited in NCBI's Gene Expression Omnibus database^23, 24^ and are accessible through GEO Series accession number GSE83386 (https://www.ncbi.nlm.nih.gov/geo/query/acc.cgi?acc=GSE83386).

### Gene expression analysis of candidate genes identified from genome-wide expression analyses

After cDNA preparation with 1 μg RNA using Transcriptor First Strand cDNA Synthesis Kit (Roche, Mannheim, Germany) in a Gradient Mastercycler (Eppendorf, Hamburg, Germany) thermocycler (10 min at 25 °C, 30 min at 55 °C, 5 min at 85 °C), gene expression was measured through real-time PCR with the QuantiTect SYBR Green PCR kit (Qiagen) in a Light Cycler 480 real-time PCR instrument (95 °C for 10 min, followed by 60 cycles of 95 °C for 10 s, and 55 °C for 15 s, 72 °C for 20 s) in technical and biological duplicates. QuantiTect Primers were purchased from Qiagen, custom-made primers from Eurofins Genomics (Ebersberg, Germany; see Supplementary Table 1). The gene expression fold-change values were calculated by the ΔΔC_T_ method using *GAPDH* as reference gene,^25^ whereas basal gene expression was indicated as ΔC_T_ values of untreated samples.

### Statistical analyses

Associations between LCL proliferation rates vs donor age and proliferation vs donor gender were calculated using Pearson's correlation and Student's *t*-test for equal variances (confirmed by Levene's test), respectively. Unpaired Student's *t*-test values were used to analyze the significance of basal gene expression differences between non-proliferating and proliferating cell lines in the edge-group approach, and to analyze basal gene expression differences between clinical subgroups in all the cell lines (clinical response after 5 and 8 weeks, and remission after 5 and 8 weeks). Data of gene expression fold-change values were analyzed by the Wilcoxon–Mann–Whitney rank-sum test. Statistical power amounts to 93.4% for EdU phenotyping experiments, to 63.57% for microarray experiments and to 99.9% for RT-PCR (PCR with reverse transcription) validation experiments with effect sizes of *r*=2 and significant levels of *α*=0.05 each. Statistical tests were calculated as two-sided and error bars are shown as standard deviations. For all the remaining applications, implemented statistics programs of the specific software (GeneSpring, STRING) were used. In general, *P*-values <0.05 were considered as significant and are reported as unadjusted unless stated otherwise. All Statistical analyses were carried out using IBM SPSS Statistics 21 (Ehningen, Germany).

## Results

**Figure 1:**
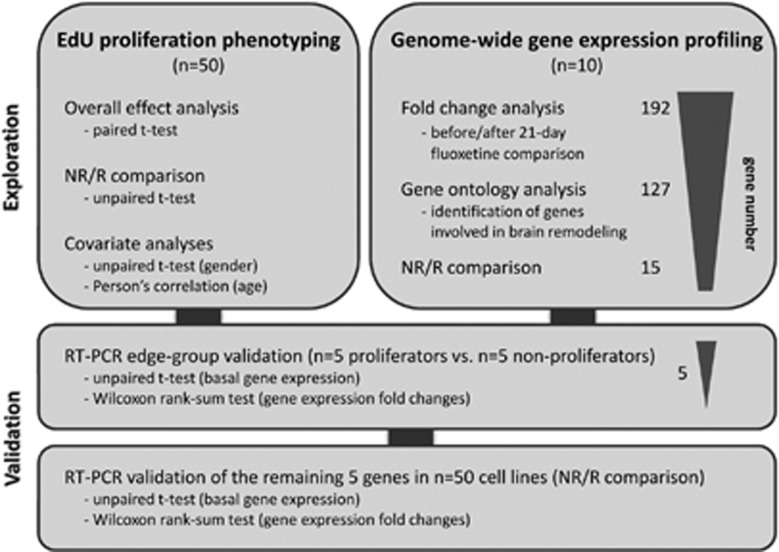
The experimental setup is divided into explorative and validative phases to identify potential gene expression biomarkers using LCLs from depressive patients. EdU, 5-ethynyl-2'-deoxyuridine; LCL, lymphoblastoid cell line; NR, non-responder; R, responder; RT-PCR, PCR with reverse transcription.

The experimental design consisted of an exploration and a validation phase (Figure 1). In the explorative phase genome-wide gene expression profiling and EdU proliferation phenotyping experiments were carried out in to identify potential gene expression biomarkers and to elucidate a possible association between individual antidepressant-induced LCL proliferation and clinical response from LCL donors, respectively. During the validation phase, both approaches were combined in an edge-group approach where the identified tentative gene expression biomarkers were evaluated in extreme cell lines from EdU phenotyping. Subsequently, the gene expression differences of the remaining candidate genes were determined between non-responder- and responder-derived cell lines.

### Genome-wide gene expression profiling

The gene expression changes following 21-day fluoxetine treatment of *n*=10 LCLs (derived from *n*=6 responders and *n*=4 non-responders representing the average patient population with different medication profiles) was measured in a genome-wide approach to characterize the late fluoxetine-induced gene expression changes and to identify potential gene expression biomarkers. Gene expression profiles were compared between untreated samples and samples treated for 21 days with 0.5 μg ml^−1^ of fluoxetine, which is similar to the average plasma concentration in fluoxetine-medicated patients. The responder- and non-responder-derived indicator cell lines were compared (characterized by rectified clinical response status, experimental EdU proliferation status and hierarchical clustering status obtained from microarray experiments), seven inversely regulated pathways were highlighted containing a total of 192 differentially expressed genes after incubation with fluoxetine (fold change >2, *P*-value <0.05). In those cell lines, STRING-based gene ontology analysis revealed 127 of the identified genes as being involved in brain remodeling (Table 2 and Supplementary Table 3). After consideration of LCL donor's individual response status (cell lines derived from *n*=6 responders vs *n*=4 non-responders), 15 genes showed different expression between responder- and non-responder-derived cell lines and, therefore, were used for further investigation and were considered as candidate genes. The gene names, fold-change differences, as well as their annotated central nervous system functions are listed in Table 3.

### Cell proliferation

**Figure 2:**
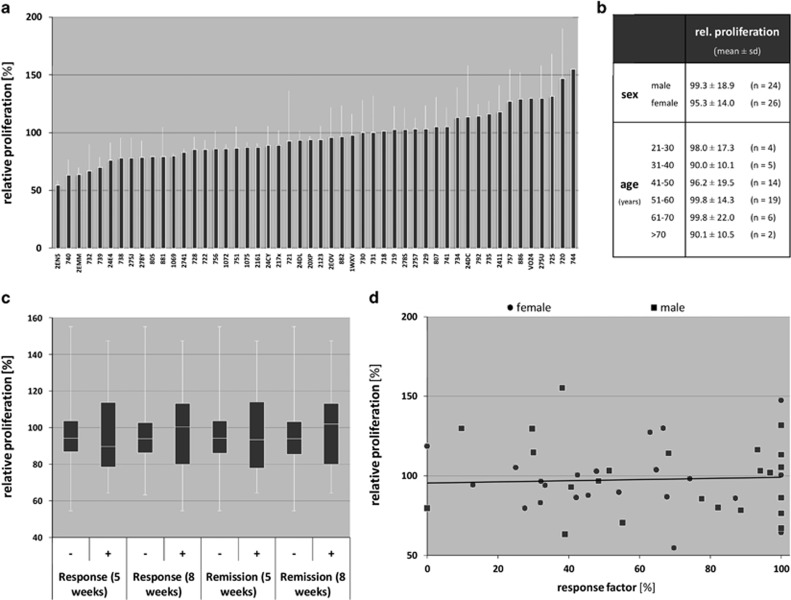
Results from EdU (5-ethynyl-2'-deoxyuridine) phenotyping experiments show individual differences between the cell lines (**a**). The covariates gender and age do not significantly influence the individual proliferation rates (**b**). Box plot analysis of EdU cell proliferation reveals no significant difference in proliferation rates after consideration of clinical response/remission status after 5 and 8 weeks of treatment (**c**). Detailed overview on the correlation between relative proliferation rates and response factor (defined as percentage change in Hamilton score compared between week 0 and 8). Each dot represents results from one cell line. As indicated by the trend line, no association between proliferation rates and response status is recognizable (**d**).

To assess the individual differences in cell proliferative effects of fluoxetine in LCLs from patients with documented clinical response status, we conducted long-term cell incubation with fluoxetine revealing large variability in relative proliferation rates ranging from 55 to 155% in comparison with untreated cells from the same donor (Figure 2a). The covariates age and gender showed no significant impact on individual proliferation rates (Figure 2b). When grouping the cell lines according to their donor's clinical response status, no significant differences between the proliferation rates of the single groups were detectable (Figure 2c). Furthermore, no association was detected between LCL proliferation rates and LCL donor's clinical response measured as percentage change in Hamilton score compared between weeks 0 and 8 (Figure 2d).

### Real-time gene expression analyses of the candidate genes

**Figure 3:**
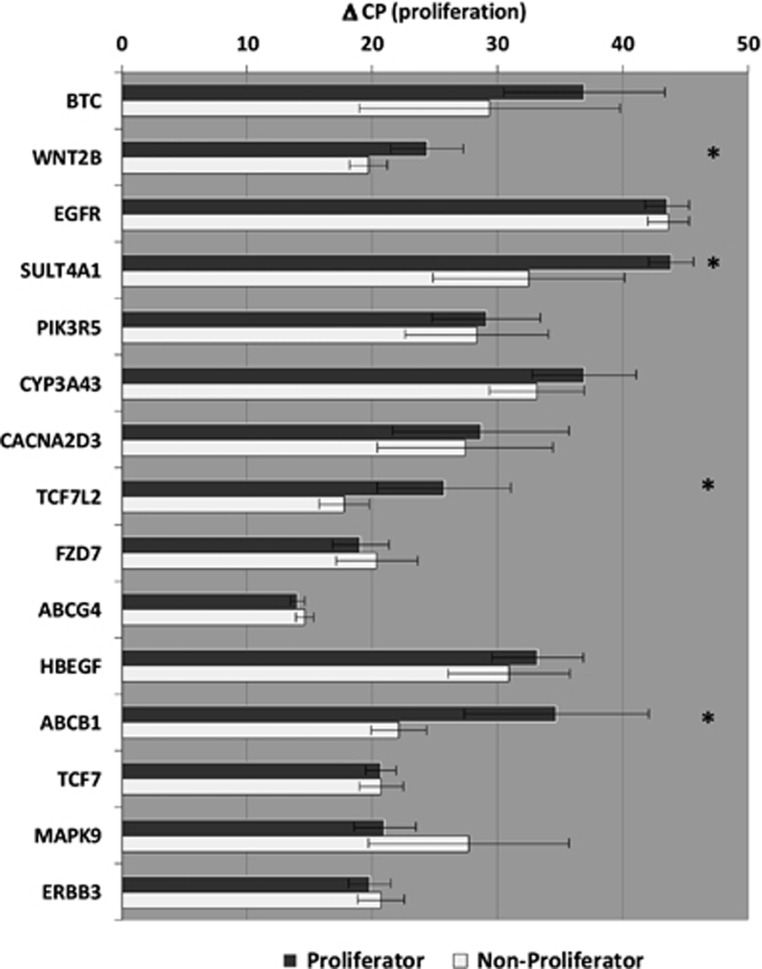
Basal gene expression of the candidate genes in an edge-group analysis from EdU (5-ethynyl-2'-deoxyuridine) phenotyping experiments.

To assess the potential of the identified candidate genes, gene expression was analyzed in an edge-group approach similar to the work of Morag *et al.*^10^ From the two phenotypic edges of EdU phenotyping (five cell lines each)—those cell lines with the most distinct fluoxetine-induced anti-proliferative and pro-proliferative effects—basal gene expression and fluoxetine-induced changes were compared. Among the 15 identified genes from our microarray experiments, the basal gene expression of four genes was significantly different from proliferator cell lines compared with non-proliferator cell lines: wingless-type MMTV integration site family, member 2B (*WNT2B*), transcription factor 7-like 2 (*TCF7L2*) sulfotransferase 4A1 (*SULT4A1*) and P-glycoprotein (*ABCB1*; Figure 3). After consideration of the LCL donor's clinical response status, no significant differences between gene expression of non-responder- and responder-derived LCLs were detectable (data not shown). In several cell lines, fluoxetine-induced gene expression changes of the above genes as well as *FZD7* (frizzled class receptor 7) were observed. Results of the fold-change analyses significantly correlated with *in vitro* proliferation of genes *WNT2B, TCF7L2* and *FZD7* (Table 4).

Changes in candidate gene expression were assessed after 21 days incubation with fluoxetine in all LCLs from EdU phenotyping experiments (*n*=50). The associations between gene expression, and both the remission and response status of LCL donors were investigated. Basal gene expression of *SULT4A1* correlated with clinical response after 5 weeks (*P*=0.029). However, basal gene expression of *SULT4A1* was low, and only detectable in 10 (*n*=4 non-responder-derived cell lines vs *n*=6 responder-derived cells) out of 50 cell lines. Furthermore, the gene expression fold-change values of *WNT2B* after treatment with fluoxetine correlated with LCL donor's clinical remission status after 5 weeks (*P*=0.025). The remaining genes *TCF7L2*, *FZD7* and *ABCB1* showed no significant correlations with clinical parameters of LCL donors (see Supplementary Table 4).

## Discussion

### Peripheral proliferation is unsuitable as surrogate marker for antidepressant response

In search of tentative functional biomarkers for antidepressant response prediction, we tested fluoxetine effects on cell proliferation in LCLs from depressed patients. Individual effects on cell proliferation have been detected after 21 days of incubation with fluoxetine. Although the *in vitro* treatment of patient-derived LCLs with fluoxetine presents high inter-individual variability regarding the LCL proliferation behavior, this phenomenon has—according to our data—no association with the patient's clinical outcome.

Our initial hypothesis was based on the assumption that antidepressants induce the proliferation of neuronal cells and therefore modulate the neural plasticity.^3^ Depressed patients show a volume reduction of depression-associated brain parts,^39^ that might be reversed by antidepressant-induced proliferation.^6^ The stimulation of neuronal stem cell proliferation in the brain is directly linked with an enhanced neuroplasticity, which eventually leads to a normalization of the depressed mood.^7^ As cerebral remodeling processes are complex and take many weeks, this phenomenon explains the observed delay (from weeks up to several months) in symptomatic improvement. Nonetheless, direct proliferative effects of antidepressants were observed (for example, by Chang *et al.*^19^) by several research groups in rodents^40, 41^ and non-human primates^42^ and by Chen *et al.*^43^ in a genetic rat model of depression. The molecular mechanisms underlying remission of depression remain unclear, although neurotrophic growth factors—like brain-derived neurotrophic factor—may have an important role during remission processes.^44^ One reason for the lack in association between clinical response and *in vitro* cell proliferation effects of *in vitro* fluoxetine treatment may be that the cell model in blood-derived LCLs is not suitable for studying brain-specific antidepressant-induced proliferative effects owing to lack of relevant neuronal pathways. Yet, several observations support a potential role of LCLs for the study of tentative biomarkers for individual variability of drug effects. For example, Morag *et al.*^10^ identified different neuronal genes (for example, *CHL1, ITGB3* or *GAP43*) as potential gene expression biomarkers to predict the response based on individual paroxetine sensitivity in LCLs. A further study using LCLs derived from depressed patients confirmed some of these genes as potential gene expression biomarkers for the prediction of individual antidepressant response.^45^ In another study, they presented a LCL-based tool to assess shared drug pathways, that was developed by comparing growth-inhibition profiles of different drug classes (including antidepressants) and can be used to categorize distinct pathways.^12^ Oved *et al.*^46^ identified potential antidepressant drug targets by genome-wide expression profiling and tentative response biomarkers in human LCLs.^9^ In addition to these studies that focused on LCLs as tools for the identification of biomarkers for depressive disorders, a few studies explored the utility of LCLs in other psychological diseases such as bipolar disorders or autism.^47, 48, 49^

Moreover, single-nucleotide polymorphisms in neuronal cell adhesion genes involved in synaptic plasticity and identified in the two latter studies performed with human LCLs, namely, *CHL1* and *ITGB3*, were recently shown to affect treatment response in depressive disorders.^50^

### Identification of potential gene expression biomarkers

Phenotyping the proliferative response of LCLs to fluoxetine (0.5 μg ml^−1^; 21 days) followed by comparative microarray-based genome-wide gene expression profiling revealed candidate genes being involved in brain remodeling processes. Genome-wide analyses of fluoxetine-induced gene expression changes in human LCLs from patients with characterized antidepressant drug response resulted in significant transcriptional regulation of 15 genes involved in neurogenesis. As microarray analyses were slightly underpowered and uncorrected for multiple testing (no false discovery rate correction), the results from the microarray analyses were further validated by RT-PCR (in the edge-group approach and—the remaining candidate genes—in all *n*=50 cell lines). By far, the strongest gene expression differences compared between responder-derived cell lines relative to non-responder-derived cell lines were obtained for betacellulin (*BTC*; with the following mean fold changes: +40.0 in responder-derived cell lines and −0.3 in non-responder-derived cell lines). *BTC* belongs to the EGF (epidermal growth factor) protein family and has been reported to stimulate neurogenesis,^26^ as well as neural stem cell proliferation and differentiation into glial- and neuronal-like cell types.^51^ *BTC* is endogenously produced in the brain, especially by blood vessels and the choroid plexus, and directly affects neuroblast differentiation and neuronal stem cell regeneration by activation of EGFR and ERBB4. It is considered a potential therapeutic agent for treating neurodegenerative diseases.^26^

Five genes (*WNT2B, TCF7L2, FZD7, SULT4A1* and *ABCB1*) were differently expressed in cell lines with the highest increase vs highest decrease in cell proliferation following 21 days fluoxetine incubation. Data analysis showed a correlation between LCL donor's clinical response (in *n*=6 responders and *n*=4 non-responders) and the basal gene expression of *SULT4A1*. Furthermore, the gene expression fold changes of *WNT2B* by fluoxetine incubation correlated with LCL donor's clinical remission. None of the remaining genes *TCF7L2*, *FZD7* and *ABCB1* showed significant correlation with clinical parameters of LCL donors.

The transcription factor TCF7L2 and the receptor FZD7 belong to the WNT signaling pathway, which has an important role for regulation of stem cell pluripotency and cell differentiation by integrating signals from other pathways and their associated signal molecules such as fibroblast growth factor^52^ and bone morphogenic protein.^53^ Both growth factors are involved in depression pathogenesis^54, 55^ and in the maintenance of adult hippocampal neurogenesis (together with brain-derived neurotrophic factor, vascular endothelial growth factor and other signaling pathways).^56^ WNT2B belongs to a family of highly conserved signal molecules involved in the regulation of neural cell growth and differentiation.^27^ Furthermore, Wnt signaling regulates adult hippocampal neurogenesis^57^ and the expansion of central nervous system progenitor cells.^58^ Moreover, it is important for synaptic function as well as for the formation of hippocampal spines.^59, 60^ A malfunction of Wnt signaling in the hippocampus by targeted knockdown is associated with decreased neurogenesis, increased depression-like behavior and various neuropsychiatric disorders.^61, 62^ Wnt glycoproteins are released by hippocampal astrocytes and take effects through gene expression activation of NeuroD and Dcx,^63, 64^ a transcription factor involved in central nervous system development and a microtubule-associated protein almost exclusively expressed in actively dividing neuronal precursor cells, respectively.^65, 66^ It has been shown that Wnt signaling is responsive to various antidepressant drugs,^67^ whereas mice with constitutively activated Wnt signaling become irresponsive to antidepressant treatments.^68^ Furthermore, a role of Wnt signaling via the fast-acting antidepressant ketamine has been proposed.^69^

Little is known so far about the brain-specific phase II metabolizing enzyme SULT4A1, but it may be involved in the metabolism of antidepressant drugs and neuroactive substances.^30^ However, as expression of *SULT4A1* was low in LCLs and only detectable in 10 out of 50 cell lines, these results should be seen with caution and warrants further analysis, of *SULT4A1* expression in brain. The transporter ABCB1 belongs to the ATP-binding cassette superfamily possessing a key role in transmembrane transport. ABCB1 is an efflux pump with a broad substrate spectrum (including a variety of antidepressant drugs as well as neurotoxic agents) transporting these substances through the blood–brain barrier into the circulatory system. The resulting ABCB1-mediated neuroprotective effect might contribute to an increased proliferation of neuronal cells. Several single-nucleotide polymorphisms in the ABCB1 gene were associated with depression severity, response status or dosage adjustments in depressive disorders indicating an involvement of ABCB1 in depression.^70, 71, 72, 73^ Fluoxetine, the antidepressant we used for *in vitro* LCL phenotyping, is a rather weak substrate of ABCB1.^74, 75^ One may speculate that the absence of a correlation between fluoxetine-induced *ABCB1* expression and clinical response could reflect the low ABCB1 substrate properties of this antidepressant drug.

## Conclusion

We measured proliferative effects of fluoxetine (21 days) in LCLs from depressed patients and analyzed association between gene expression changes of the genes identified by microarray analysis and cell proliferation. Three of the 15 genes identified from genome-wide analyses showed significant associations with cell proliferative behavior. Furthermore, for the gene expression of two candidate genes, *SULT4A1* and *WNT2B*, we observed correlations between LCL donor's clinical response and remission, respectively. These genes are involved in the metabolism of antidepressants and neuroactive agents, and in neural cell proliferation and differentiation, respectively. Further studies should follow to elucidate a connection between cellular proliferation effects and clinical antidepressant response. The candidate genes reported here should be further examined for their pharmacogenetic variability and their role in remission from depression using longitudinal blood samples from major depression patients, as well as brain tissues from animal models for depression.

## Figures and Tables

**Table 1:** Characteristics of the MARS LCL study cohort with significant group differences indicated

	*Responder*	*Non-responder*	*Significance (*P*-value)*
*Gender*			
Male	*n*=14	*n*=10	NS
Female	*n*=11	*n*=15	
			
Age (years)	48.3±12.2	51.6±11.4	NS
			
*Hamilton score*			
Week 0	28.6±6.3	25.5±8.2	NS
Week 5	8.3±6.4	19.7±5.4	0.000
Week 8	4.8±5.0	18.7±5.1	0.000
			
*Number of different antidepressants*			
1	*n*=11	*n*=6	
2	*n*=13	*n*=8	
3	*n*=1	*n*=9	
4	*n*=0	*n*=2	
Mean	*n*=1.6±0.6	*n*=2.3±0.9	0.003

Abbreviations: LCL, lymphoblastoid cell line; MARS, Munich Antidepressant Response Signature; NS, not significant.

**Table 2:** Gene ontology terms of the 192 differentially expressed genes found by microarray experiments

*GO term*	P*-value*	*Corrected* P*-value*	n
Neuron differentiation	5.98e−27	7.54e−23	44
Generation of neurons	2.03e−25	8.22e−22	47
Neuron projection development	2.24e−25	8.22e−22	37
Axon development	3.00e−25	8.22e−22	34
Neuron projection morphogenesis	1.08e−24	1.94e−21	34
Axonogenesis	1.91e−23	2.68e−20	32
Neuron development	6.92e−23	7.27e−20	37
Canonical Wnt signaling pathway	1.04e−22	9.41e−20	18
Neurogenesis	2.10e−22	1.53e−19	45

Abbreviation: GO, gene ontology.

The *P*-values were calculated by STRING web-tool and indicated as uncorrected *P*-values and Benjamini–Hochberg corrected *P*-values (*n* is the number of identified genes being involved in particular GO terms). The GO terms are arranged in terms of increasing *P*-values.

**Table 3:** Comparison of mean gene expression levels between responder and non-responder cell lines (*n=*10) and their annotated gene functions

*Gene (Entrez ID)*	*Mean FC difference (responder vs non-responder)*	*CNS function*
*BTC* (685)	40.30	Stimulation of cell proliferation and neurogenesis^26^
*WNT2B* (7482)	26.20	Regulation of pro-neural genes^27^
*EGFR* (1956)	18.40	Neural progenitor cells proliferation and migration^28^
*CYP3A43* (64816)	6.90	Antipsychotic metabolism^29^
*PIK3R5* (23533)	6.70	Unknown
*SULT4A1* (25830)	6.20	Brain-specific sulfate conjugation of drugs and neurotransmitters^30^
*FZD7* (8324)	5.40	Receptor for Wnt proteins in brain^31^
*CACNA2D3* (55799)	5.30	Possible role in long-term antidepressants action^32^
*TCF7L2* (6934)	4.73	Transcription factor in Wnt pathway^31^
*ABCG4* (64137)	4.10	Regulation of lipid homeostasis in neurons and astrocytes^33, 34^
*TCF7* (6932)	3.60	Transcription factor in Wnt pathway^31^
*HBEGF* (1839)	3.50	Neurogenesis and astrocytes proliferation^35^
*MAPK9* (5601)	2.50	Mediates apoptosis in dopaminergic brain areas^36^
*ABCB1* (5243)	2.45	Export of neurotoxic agents in blood–brain barrier^37^
*ERBB3* (2065)	2.00	Nervous system development^38^

Abbreviations: CNS, central nervous system; FC, fold change.

Full gene names are listed in Supplementary Table 1.

**Table 4:** Fold-change values of the candidate genes after treatment with fluoxetine

*Cell line*	WNT2B	SULT4A1	TCF7L2	FZD7	ABCB1
*Proliferators*					
744	10.4	NE	>1000	−60.4	>10 000
720	0.01	175.0	481.6	−2.8	−1.9
725	695.2	>10 000	>10 000	<−1000	>10 000
275U	29.5	NE	6.9	−119.8	>10 000
VO24	>10 000	NE	>10 000	−306.5	>10 000
Mean	>1000	>10 000	>10 000	<−1000	>10 000
					
*Non-proliferators*					
2EN5	1.4	NE	1.7	2.0	1.1
740	−0.7	NE	0.3	−6.5	−5.6
2EMM	−63.7	NE	6.8	44.8	0.3
732	−1.6	−0.3	1.8	1.9	0.1
739	2.6	−3.6	2.7	2.5	1.4
Mean	−12.4	−1.9	2.6	8.9	-0.5

*P*-value	0.032	0.333	0.008	0.016	0.095

Abbreviation: NE, not estimable.
