# Supplementary material for: Proliferation rates and gene expression profiles in human lymphoblastoid cell lines from patients with depression characterized in response to antidepressant drug therapy
Source: Transl Psychiatry. 2016 Nov 15;6(11):e950–. doi: 10.1038/tp.2016.185 (PMC5314111; doi:10.1038/tp.2016.185)
Supplement: Supplementary Information [file tp2016185x1.docx]

**Supplement**

Supplementary Table 1: Primers used for real-time PCR experiments.

| **gene** | **full gene name** | **Assay name or sequence** |
| --- | --- | --- |
| *BTC* | betacellulin | Hs_BTC_1_SG |
| *EGFR* | epidermal growth factor receptor | Hs_EGFR_vb.1_SG |
| *WNT2B* | wingless-type MMTV integration site family, member 2B | Hs_WNT2B_va.1_SG |
| *SULT4A1* | sulfotransferase family 4A, member 1 | Hs_SULT4A1_1_SG |
| *ABCB1* | ABC-Transporter B1 (P-glycoprotein) | Hs_ABCB1_1_SG |
| *PIK3R5* | phosphoinositide-3-kinase, regulatory subunit 5 | HS_PIK3R5_1_SG |
| *CYP3A43* | cytochrome P450 3A43 | HS_CYP3A43_1_SG |
| *HBEGF* | heparin-binding EGF-like growth factor | HS_HBEGF_1_SG |
| *TCF7L2* | transcription factor 7-like 2 | Hs_TCF7L2_1_SG |
| *ERBB3* | epidermal growth factor receptor 3 | Hs_ERBB3_vb.1_SG |
| *FZD7* | frizzled homolog 7 | Fwd: CCTTCCCCTTCTCATGCCC  Rev: CAGCCCGACAGGAAGATGAT |
| *MAPK9* | mitogen-activated protein kinase 9 | Hs_MAPK9_va.1_SG |
| *PRKCA* | protein kinase C, alpha | Hs_PRKCA_1_SG |
| *TCF7* | transcription factor 7 | Hs_TCF7_va.1_SG |
| *CACNA2D3* | calcium channel, voltage-dependent, alpha 2/delta subunit 3 | Hs_CACNA2D3_1_SG |
| *GAPDH* | Glyceraldehyde 3-phosphate dehydrogenase | Hs_CACNA2D3_1_SG |

Supplementary Figure 1: Clinical data of microarray cell lines


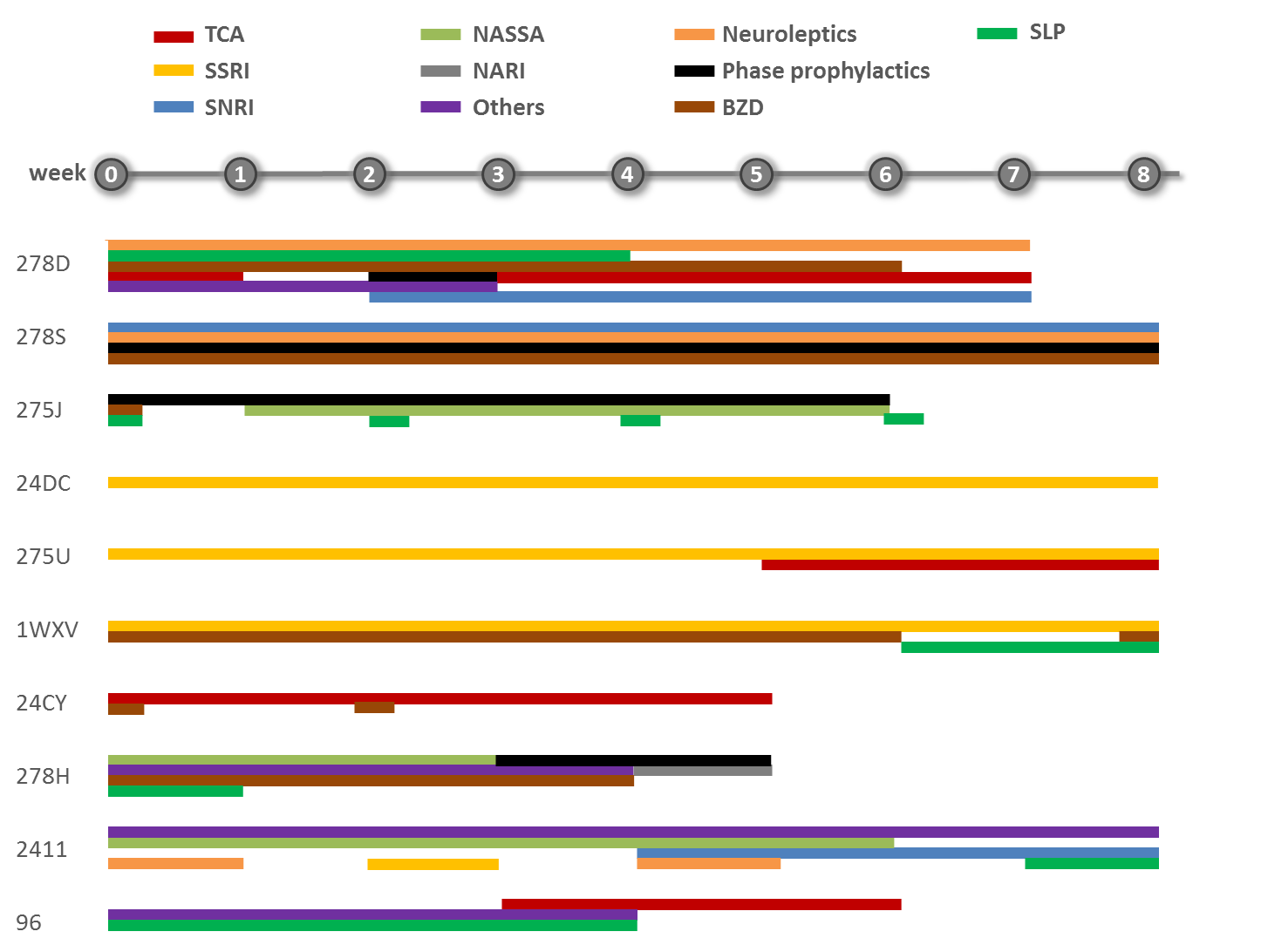


Abbreviations: TCA (tricyclic antidepressants), SSRI (selective serotonin reuptake inhibitors), SNRI (serotonin-norepinephrine reuptake inhibitors), NASSA (noradrenergic and specific serotonergic antidepressant), NARI (noradrenalin-reuptake-inhibitor), BZD (benzodiazepines), SLP (sleep medication)

Supplementary Table 2: Clinical Data Overview

Abbreviations: f (female), m (male), TCA (tricyclic antidepressants), SSRI (selective serotonin reuptake inhibitors), SNRI (serotonin-norepinephrine reuptake inhibitors), NASSA (noradrenergic and specific serotonergic antidepressant), NARI (noradrenalin-reuptake-inhibitor), SSRE (selective serotonin reuptake enhancer)

Supplementary Table 3: Microarray results (fold changes) of identified genes being involved GO terms of brain remodeling processes. Mean values and standard deviations (sd) of responder (R) and non-responder (NR) derived cell lines are indicated as well as p-values calculated by Wilcoxon-Mann-Whitney rank-sum test. Significant p-values are in bold.

| **genes** | | **cell lines** | | | | | | | | | | **mean (sd)** | | | | | | |
| --- | --- | --- | --- | --- | --- | --- | --- | --- | --- | --- | --- | --- | --- | --- | --- | --- | --- | --- |
| **ProbeName** | **Symbol** | **1WXV** | **2411** | **24DC** | **275J** | **275U** | **278D** | **278H** | **278S** | **96** | **CY24** | **R** | | | **NR** | | | **p-value** |
| A_24_P282416 | ABL1 | -1,1 | -1,2 | -1,5 | -2,5 | -2,1 | 1,1 | -1,3 | 1,2 | -1,9 | 1,0 | -0,8 | ± | 1,6 | -0,9 | ± | 1,3 | 1,00 |
| A_23_P135769 | ACTB | 1,1 | -1,5 | 2,1 | 2,3 | 5,0 | 2,1 | -1,0 | -1,3 | -1,7 | -2,0 | 1,9 | ± | 2,0 | -1,6 | ± | 0,4 | **0,02** |
| A_23_P105957 | ACTN1 | -1,1 | -1,5 | -2,1 | -2,4 | -1,1 | 1,4 | -1,3 | -1,3 | -2,4 | -2,0 | -1,1 | ± | 1,3 | -1,8 | ± | 0,5 | 0,35 |
| A_23_P101655 | ACTN4 | -1,2 | -1,8 | -2,2 | -3,0 | 1,2 | 1,6 | -1,6 | -1,3 | -2,7 | -2,2 | -0,8 | ± | 1,9 | -2,1 | ± | 0,5 | 0,26 |
| A_33_P3408983 | APC | -1,2 | 1,4 | 2,5 | 4,4 | 1,7 | 1,2 | 1,7 | 1,7 | 2,4 | 1,4 | 1,7 | ± | 1,8 | 1,7 | ± | 0,5 | 1,00 |
| A_23_P73511 | ARAF | 1,0 | -1,0 | -2,0 | -2,6 | -1,1 | 1,4 | -1,6 | -1,4 | -2,2 | -1,6 | -0,8 | ± | 1,6 | -1,6 | ± | 0,5 | 0,61 |
| A_24_P298027 | AXIN2 | 4,2 | 1,2 | 2,6 | 1,7 | 1,2 | 1,1 | -1,3 | -1,1 | -1,9 | 1,1 | 1,6 | ± | 1,7 | -0,2 | ± | 1,6 | 0,11 |
| A_23_P148015 | AXIN2 | 1,2 | 1,1 | 1,0 | -1,6 | -5,8 | -3,4 | 1,1 | -1,1 | -1,0 | 1,4 | -1,6 | ± | 2,7 | 0,6 | ± | 1,1 | 0,11 |
| A_33_P3398526 | BCL2L11 | -1,1 | -1,2 | 1,5 | 3,0 | 2,6 | -1,1 | 1,1 | -1,3 | -1,4 | -1,1 | 0,6 | ± | 2,0 | -0,6 | ± | 1,2 | 0,11 |
| A_33_P3251932 | BCL2L11 | -1,2 | -1,6 | -2,1 | -1,7 | -1,7 | -1,3 | -1,2 | -1,4 | -2,0 | -1,6 | -1,6 | ± | 0,3 | -1,6 | ± | 0,3 | 0,26 |
| A_23_P135722 | BTC | 1,8 | -1,1 | 107,1 | 103,2 | 30,6 | -1,1 | 2,1 | -1,9 | -1,0 | -1,3 | 40,0 | ± | 52,0 | -0,3 | ± | 1,6 | 0,91 |
| A_23_P373031 | CACNA1C | -1,1 | -1,4 | 2,7 | 4,2 | 2,1 | 1,1 | -1,0 | -1,4 | -1,3 | -1,5 | 1,3 | ± | 2,2 | -1,3 | ± | 0,2 | 0,35 |
| A_33_P3297580 | CACNA1C | 4,6 | -1,1 | -2,1 | 1,0 | -4,0 | -2,6 | -4,3 | 1,2 | 1,2 | -1,5 | -0,3 | ± | 3,2 | -1,4 | ± | 2,2 | 0,07 |
| A_33_P3825869 | CACNA1C | -7,1 | -1,0 | -3,0 | 1,4 | -3,8 | -1,9 | 1,3 | 1,2 | -5,4 | 1,2 | -2,2 | ± | 3,2 | -1,0 | ± | 3,1 | 0,76 |
| A_23_P365767 | CACNA1D | -1,2 | 1,6 | -1,1 | 2,2 | 3,6 | -1,2 | 1,1 | 1,1 | -2,2 | -2,0 | 0,6 | ± | 2,1 | -0,4 | ± | 2,0 | 0,61 |
| A_23_P148327 | CACNA1F | 1,0 | 1,0 | 1,3 | -1,1 | -6,4 | -1,5 | 1,0 | -1,1 | -1,6 | 1,6 | -1,3 | ± | 2,8 | 0,5 | ± | 1,5 | 0,35 |
| A_23_P85765 | CACNA1S | 1,1 | -1,2 | -4,1 | -2,4 | -1,6 | -1,1 | -1,4 | -1,3 | 1,0 | 1,1 | -1,6 | ± | 1,7 | -0,1 | ± | 1,4 | 0,48 |
| A_24_P91165 | CACNB1 | -1,2 | 1,3 | 1,5 | 2,9 | 3,0 | 1,1 | -1,2 | -1,1 | -1,8 | -1,3 | 1,0 | ± | 1,8 | -0,7 | ± | 1,4 | 0,26 |
| A_33_P3285277 | CACNB2 | 1,5 | -2,3 | -1,1 | 3,9 | 2,8 | -1,0 | -1,1 | 1,1 | 1,4 | -1,1 | 1,2 | ± | 2,0 | -0,8 | ± | 1,5 | 0,07 |
| A_33_P3363271 | CACNB2 | 1,2 | 1,0 | -1,1 | -2,3 | -2,8 | -1,2 | -1,3 | 1,2 | 1,3 | -1,3 | -0,8 | ± | 1,7 | -0,1 | ± | 1,4 | 0,11 |
| A_33_P3221989 | CACNB4 | 1,1 | 1,4 | 1,1 | 1,7 | -2,1 | -1,2 | -1,0 | 1,4 | 2,3 | 3,8 | 0,3 | ± | 1,6 | 1,6 | ± | 2,0 | 0,76 |
| A_23_P300056 | CDC42 | 1,2 | 1,1 | -1,8 | -3,1 | -2,2 | -1,5 | -1,0 | 1,3 | 1,5 | 1,0 | -1,0 | ± | 1,8 | 0,6 | ± | 1,1 | 0,17 |
| A_24_P81841 | CDKN1B | -1,1 | 1,4 | -1,2 | -1,7 | -2,2 | -1,1 | 1,3 | 1,3 | 2,2 | 1,9 | -1,0 | ± | 1,2 | 1,7 | ± | 0,4 | 0,26 |
| A_24_P270814 | CRK | 1,1 | -1,1 | -2,5 | -4,7 | -3,6 | 1,6 | -1,3 | -1,2 | -1,8 | -1,4 | -1,5 | ± | 2,5 | -1,4 | ± | 0,3 | **0,01** |
| A_33_P3265180 | CSNK1E | -1,0 | -1,6 | 1,4 | 1,4 | 2,0 | 1,1 | -1,2 | -1,1 | -2,0 | -1,9 | 0,6 | ± | 1,4 | -1,7 | ± | 0,4 | 0,91 |
| A_24_P103922 | CTBP2 | 2,4 | -1,1 | 2,5 | 3,4 | 2,7 | 1,2 | 1,2 | -1,2 | -1,0 | -1,1 | 1,8 | ± | 1,7 | -0,5 | ± | 1,1 | **0,01** |
| A_23_P63897 | CTBP2 | -1,1 | 1,4 | 1,6 | 2,0 | 2,3 | 1,1 | 1,2 | -1,1 | 2,4 | 1,5 | 0,8 | ± | 1,5 | 1,6 | ± | 0,6 | 0,11 |
| A_23_P58647 | CTNNA1 | 1,1 | 1,1 | -1,4 | -2,2 | -2,0 | -1,1 | -1,1 | 1,2 | 1,0 | 1,0 | -0,7 | ± | 1,5 | 0,5 | ± | 1,1 | 0,61 |
| A_33_P3209716 | CTNND1 | -1,1 | -1,2 | 7,0 | 4,7 | 2,9 | 1,3 | -1,3 | -1,2 | -1,5 | -1,7 | 2,3 | ± | 3,2 | -1,4 | ± | 0,2 | 0,48 |
| A_23_P251316 | CTNND1 | 1,1 | 1,0 | -1,3 | -2,7 | -2,3 | -1,1 | 1,0 | 1,1 | 1,1 | 1,5 | -0,9 | ± | 1,6 | 1,1 | ± | 0,2 | **0,01** |
| A_24_P225961 | DAG1 | -1,2 | -1,0 | -2,8 | -2,3 | -2,3 | 1,1 | -1,3 | -1,4 | -1,5 | -1,3 | -1,5 | ± | 1,4 | -1,3 | ± | 0,2 | 0,17 |
| A_33_P3344579 | DLD | 1,0 | -1,1 | -1,6 | -1,9 | -2,1 | 1,2 | -1,0 | 1,1 | 1,2 | 1,2 | -0,4 | ± | 1,6 | 0,1 | ± | 1,3 | 0,48 |
| A_24_P185854 | DMD | 1,2 | 1,3 | 1,0 | -2,0 | -3,6 | -1,5 | 1,1 | 1,0 | 1,7 | 1,8 | -0,7 | ± | 2,0 | 1,5 | ± | 0,3 | 0,35 |
| A_23_P347432 | DVL1 | -1,2 | -1,7 | -2,4 | -1,8 | 1,1 | 1,2 | -1,4 | -1,3 | -2,0 | -1,5 | -0,7 | ± | 1,5 | -1,7 | ± | 0,3 | **0,02** |
| A_33_P3351944 | EGFR | 1,1 | -1,1 | 69,1 | 24,2 | 14,5 | -1,2 | -1,7 | -1,1 | -1,1 | 1,2 | 17,8 | ± | 27,1 | -0,7 | ± | 1,3 | 0,35 |
| A_33_P3386099 | ELK1 | -1,5 | -1,1 | 4,0 | 4,1 | 2,9 | 1,4 | -1,5 | -1,2 | -1,3 | 1,1 | 1,6 | ± | 2,5 | -0,7 | ± | 1,2 | 0,26 |
| A_23_P40693 | EP300 | 1,0 | 1,1 | -1,6 | -2,2 | -2,1 | 1,4 | -1,1 | 1,1 | 1,2 | 1,1 | -0,4 | ± | 1,7 | 0,5 | ± | 1,1 | 0,17 |
| A_23_P89249 | ERBB2 | 1,0 | -1,1 | 1,7 | 2,2 | 2,3 | -1,4 | 1,1 | 1,3 | 1,1 | -1,3 | 1,2 | ± | 1,4 | -0,1 | ± | 1,3 | 0,35 |
| A_33_P3292596 | ERBB2 | 1,2 | -1,0 | 1,4 | 2,1 | 2,4 | -1,2 | -1,1 | 1,4 | -1,1 | -2,4 | 1,2 | ± | 1,3 | -1,4 | ± | 0,6 | 0,26 |
| A_23_P349416 | ERBB3 | 1,0 | -1,2 | -3,2 | -1,1 | -1,6 | 1,2 | 3,6 | -1,2 | 1,0 | 1,1 | -0,8 | ± | 1,7 | 1,1 | ± | 2,0 | 0,07 |
| A_33_P3211558 | ERBB4 | -1,1 | -1,0 | -1,0 | 1,2 | 4,3 | 1,2 | 1,1 | -1,2 | 1,6 | 1,4 | 0,6 | ± | 2,1 | 0,8 | ± | 1,2 | 0,26 |
| A_32_P183765 | ERBB4 | -4,3 | -1,0 | -2,0 | -1,3 | -1,1 | -4,5 | 1,8 | 1,0 | 1,8 | 1,3 | -2,0 | ± | 2,1 | 0,9 | ± | 1,3 | 0,48 |
| A_23_P41344 | EREG | -1,5 | -1,3 | 1,1 | -2,3 | 2,2 | 2,0 | 1,3 | 1,6 | -1,0 | 2,2 | 0,5 | ± | 1,9 | 0,3 | ± | 1,7 | **0,02** |
| A_23_P161624 | FOSL1 | -1,1 | -1,6 | -1,1 | 1,6 | 2,7 | 1,2 | -1,7 | -1,1 | -2,8 | -1,5 | 0,4 | ± | 1,7 | -1,9 | ± | 0,6 | 0,91 |
| A_24_P22079 | FOXO1 | 1,1 | 1,5 | 1,0 | -2,1 | -2,4 | 1,6 | -1,2 | 1,0 | 1,1 | 1,1 | 0,0 | ± | 1,8 | 0,7 | ± | 1,2 | **0,01** |
| A_33_P3261173 | FRAT1 | -1,3 | -1,4 | 1,3 | -1,3 | 2,4 | -1,0 | -3,3 | -1,3 | -1,0 | 2,1 | -0,2 | ± | 1,6 | -0,9 | ± | 2,2 | 0,48 |
| A_33_P3367899 | FRAT1 | -1,0 | -1,1 | -2,9 | -1,6 | -1,1 | -1,2 | 2,3 | 1,2 | 1,4 | 2,7 | -1,1 | ± | 1,3 | 1,3 | ± | 1,7 | 0,48 |
| A_24_P38276 | FZD1 | -1,0 | 2,0 | -4,0 | -2,3 | -2,8 | 1,4 | 1,0 | 1,1 | 1,5 | 1,8 | -1,3 | ± | 2,1 | 1,6 | ± | 0,4 | **0,04** |
| A_23_P203972 | FZD10 | 3,4 | -1,6 | -2,3 | -2,8 | 1,2 | -2,8 | 8,1 | 1,3 | 1,7 | 1,2 | -0,3 | ± | 2,7 | 2,4 | ± | 4,1 | **0,04** |
| A_23_P141362 | FZD2 | -1,5 | -1,4 | -6,1 | -4,1 | -1,4 | -1,2 | -1,8 | 1,0 | -1,5 | -1,5 | -2,2 | ± | 2,5 | -1,6 | ± | 0,2 | 0,26 |
| A_23_P347468 | FZD3 | -1,3 | 1,4 | -2,6 | -2,7 | -2,6 | -1,3 | 1,8 | 1,3 | 2,0 | 2,6 | -1,5 | ± | 1,6 | 1,9 | ± | 0,5 | 0,76 |
| A_23_P64617 | FZD4 | -5,8 | -1,1 | -1,1 | -1,4 | -2,2 | 1,3 | 1,1 | -1,0 | -6,1 | 3,2 | -1,7 | ± | 2,3 | -0,7 | ± | 4,0 | **0,01** |
| A_23_P108437 | FZD5 | 1,1 | 1,5 | -2,1 | -2,6 | -2,9 | -1,0 | -1,2 | 1,1 | 1,0 | 1,0 | -1,1 | ± | 1,8 | 0,6 | ± | 1,2 | 0,61 |
| A_33_P3409392 | FZD6 | 1,2 | 1,2 | -1,5 | -1,7 | -2,3 | -1,3 | -1,0 | 1,2 | 1,3 | 1,4 | -0,8 | ± | 1,5 | 0,7 | ± | 1,2 | 0,35 |
| A_23_P209449 | FZD7 | 1,1 | 2,0 | -1,8 | -2,3 | -15,2 | -1,3 | 1,3 | -1,0 | 1,8 | 3,0 | -3,4 | ± | 5,9 | 2,0 | ± | 0,7 | 0,07 |
| A_23_P59613 | FZD9 | -1,3 | -2,1 | 1,0 | -1,1 | 2,5 | 1,2 | -2,0 | 1,1 | 1,1 | -1,8 | 0,6 | ± | 1,5 | -1,2 | ± | 1,5 | **0,01** |
| A_23_P34093 | G6PD | -1,3 | -1,4 | -3,3 | -3,7 | -1,3 | 1,5 | -1,9 | -1,4 | -2,5 | -1,5 | -1,6 | ± | 1,9 | -1,8 | ± | 0,5 | 0,07 |
| A_23_P335239 | GAB1 | -1,2 | -1,0 | 2,9 | 1,6 | -1,9 | 1,5 | 1,0 | 1,7 | 1,3 | -1,0 | 0,8 | ± | 1,9 | 0,1 | ± | 1,3 | 0,48 |
| A_24_P55295 | GJA1 | -1,1 | -1,0 | -2,7 | -1,0 | 18,3 | -1,4 | 1,0 | 1,4 | -1,1 | -1,4 | 2,2 | ± | 8,0 | -0,6 | ± | 1,1 | 0,48 |
| A_24_P407717 | GRB2 | -1,1 | -1,1 | -1,9 | -3,0 | -2,0 | 1,0 | -1,1 | -1,2 | -1,5 | -1,2 | -1,4 | ± | 1,3 | -1,2 | ± | 0,2 | 1,00 |
| A_32_P31618 | GSR | 1,0 | -1,0 | -2,2 | -2,3 | -3,5 | -1,1 | 1,0 | -1,2 | -1,2 | -1,2 | -1,5 | ± | 1,5 | -0,6 | ± | 1,1 | 0,48 |
| A_24_P140608 | HBEGF | -1,3 | 1,0 | -10,4 | -7,3 | -2,6 | 1,4 | 1,3 | -1,2 | -1,7 | -1,0 | -3,6 | ± | 4,4 | -0,1 | ± | 1,5 | 0,26 |
| A_33_P3353791 | ITGA1 | 1,2 | 1,3 | -1,2 | -1,7 | -7,0 | -1,3 | -1,2 | 1,1 | -1,1 | -1,3 | -1,5 | ± | 3,0 | -0,6 | ± | 1,3 | 0,26 |
| A_33_P3231447 | ITGA6 | -1,2 | 1,9 | -2,3 | -1,7 | -1,3 | 1,5 | 1,2 | 1,7 | 1,1 | 1,6 | -0,5 | ± | 1,7 | 1,4 | ± | 0,4 | 0,48 |
| A_23_P210176 | ITGA6 | -1,1 | -1,0 | 2,5 | 1,2 | -1,4 | -1,7 | -13,5 | 2,5 | -1,1 | 1,3 | 0,3 | ± | 2,0 | -3,6 | ± | 6,7 | 0,17 |
| A_23_P50907 | ITGAV | 1,0 | 1,4 | -1,3 | -1,9 | -2,1 | -1,4 | 1,1 | 1,2 | 1,5 | 1,6 | -0,8 | ± | 1,5 | 1,4 | ± | 0,2 | 0,61 |
| A_23_P104199 | ITGB1 | 1,1 | 1,3 | 2,1 | 1,4 | 2,2 | 1,3 | -1,1 | -1,3 | 1,1 | 1,0 | 1,1 | ± | 1,3 | 0,6 | ± | 1,1 | **0,02** |
| A_24_P318656 | ITGB3 | -1,1 | -1,0 | 2,3 | 5,0 | 1,8 | 1,6 | 1,1 | -1,1 | 1,5 | -1,3 | 1,4 | ± | 2,3 | 0,1 | ± | 1,4 | 0,26 |
| A_33_P3377364 | ITGB4 | 1,2 | -3,6 | -2,8 | -1,7 | 1,2 | -1,3 | 1,1 | -1,3 | -1,1 | 1,6 | -0,8 | ± | 1,7 | -0,5 | ± | 2,4 | 0,26 |
| A_33_P3323298 | JUN | 1,1 | 1,5 | 1,1 | -2,3 | -3,6 | -1,4 | -1,1 | -1,0 | 1,9 | 2,5 | -1,0 | ± | 1,9 | 1,2 | ± | 1,6 | 0,76 |
| A_33_P3309984 | KHK | 1,1 | 1,9 | -3,3 | -2,1 | -3,0 | 1,7 | -1,5 | 1,1 | 1,5 | 1,1 | -0,8 | ± | 2,3 | 0,8 | ± | 1,6 | 0,07 |
| A_23_P70719 | LAMA2 | -1,3 | -1,2 | 2,3 | 1,6 | 1,5 | -1,3 | 1,2 | -1,3 | 1,4 | -1,2 | 0,3 | ± | 1,7 | 0,0 | ± | 1,5 | 0,26 |
| A_24_P20630 | LEF1 | 1,2 | 2,6 | -2,1 | -1,0 | -1,4 | -1,9 | 1,0 | 1,9 | 2,1 | 2,0 | -0,6 | ± | 1,7 | 1,9 | ± | 0,7 | 1,00 |
| A_23_P1505 | LRP5 | -1,4 | -2,5 | -1,4 | 1,0 | 2,6 | 1,2 | -1,3 | -1,5 | -2,8 | -2,7 | 0,1 | ± | 1,7 | -2,3 | ± | 0,7 | **0,04** |
| A_33_P3389872 | LRRK2 | -1,1 | 1,0 | -2,1 | -6,4 | -1,4 | -1,0 | 1,2 | 1,0 | 3,6 | 2,3 | -1,8 | ± | 2,5 | 2,0 | ± | 1,2 | 0,07 |
| A_33_P3369058 | LRRK2 | 1,3 | -1,0 | 1,0 | -2,6 | -2,2 | -1,1 | 1,3 | 1,4 | 1,7 | 1,5 | -0,4 | ± | 1,8 | 0,9 | ± | 1,3 | **0,01** |
| A_23_P20248 | MAP2K1 | -1,1 | -1,2 | -1,4 | -2,9 | -2,3 | 1,0 | -1,2 | 1,0 | -1,5 | -1,3 | -0,9 | ± | 1,7 | -1,3 | ± | 0,1 | 0,11 |
| A_33_P3236651 | MAP2K7 | 1,1 | -1,8 | 1,1 | 1,5 | 3,0 | 1,2 | -1,4 | 1,1 | -1,9 | -2,0 | 1,5 | ± | 0,8 | -1,8 | ± | 0,2 | 1,00 |
| A_33_P3254216 | MAPK10 | -1,6 | 1,1 | -2,0 | -1,4 | -1,6 | -1,4 | -3,6 | -1,1 | -1,1 | -1,3 | -1,5 | ± | 0,3 | -1,2 | ± | 1,9 | **0,01** |
| A_24_P286898 | MAPK8 | 1,1 | 1,4 | -1,8 | -2,0 | -3,1 | -1,1 | -1,2 | 1,3 | 1,2 | 1,9 | -0,9 | ± | 1,8 | 0,8 | ± | 1,4 | 0,35 |
| A_33_P3250289 | MAPK8 | 1,1 | -1,4 | -1,0 | -1,2 | -2,8 | -1,3 | 1,3 | -1,0 | -1,0 | 1,3 | -1,0 | ± | 1,3 | 0,0 | ± | 1,5 | 0,11 |
| A_33_P3400192 | MAPK9 | 1,1 | 2,3 | -1,2 | -1,7 | -3,5 | 1,0 | 1,2 | 1,0 | 2,0 | 2,6 | -0,5 | ± | 1,9 | 2,0 | ± | 0,6 | 0,35 |
| A_23_P52761 | MMP7 | -1,8 | 1,1 | -1,4 | -1,9 | -3,3 | -2,8 | -1,1 | 1,1 | -1,4 | 1,7 | -1,7 | ± | 1,5 | 0,1 | ± | 1,5 | **0,01** |
| A_33_P3245163 | MYC | 1,0 | -2,4 | -2,1 | -2,0 | -2,5 | -6,3 | 1,2 | 1,3 | 1,0 | -1,3 | -1,8 | ± | 2,8 | -0,3 | ± | 1,8 | 0,07 |
| A_23_P204640 | NANOG | -2,2 | -3,1 | -1,4 | -1,6 | -2,1 | -2,3 | -1,5 | 2,0 | -4,2 | 1,3 | -1,3 | ± | 1,7 | -1,9 | ± | 2,4 | 0,48 |
| A_23_P255785 | NCK1 | 1,1 | 1,4 | -1,3 | -2,4 | -2,5 | -1,2 | 1,0 | 1,0 | 1,2 | 1,2 | -0,9 | ± | 1,6 | 1,2 | ± | 0,2 | 0,61 |
| A_33_P3252954 | NKD2 | 1,3 | 1,3 | 2,9 | 3,7 | 2,4 | -1,0 | 1,0 | 1,2 | 1,7 | 1,3 | 1,8 | ± | 1,7 | 1,3 | ± | 0,3 | **0,02** |
| A_23_P315815 | NRG1 | 1,5 | -1,3 | -2,7 | 1,8 | -1,5 | -2,1 | 1,1 | -1,5 | -1,0 | 1,5 | -0,8 | ± | 1,9 | 0,1 | ± | 1,5 | 0,61 |
| A_33_P3395513 | NRG2 | 1,2 | 1,0 | 2,6 | 4,6 | 2,1 | 1,6 | 1,8 | -1,2 | 1,5 | 1,2 | 1,8 | ± | 1,9 | 1,4 | ± | 0,3 | 0,35 |
| A_23_P334727 | NRG4 | -1,4 | 1,2 | 1,4 | 2,4 | 3,2 | -1,7 | 1,2 | 1,1 | 1,3 | 1,4 | 0,8 | ± | 2,0 | 1,3 | ± | 0,1 | 0,35 |
| A_23_P59138 | POU5F1 | 1,1 | 1,3 | 3,2 | 5,4 | 6,2 | -1,5 | 1,7 | 1,6 | 1,9 | -1,1 | 2,7 | ± | 2,9 | 0,9 | ± | 1,4 | 0,91 |
| A_24_P252130 | PPARD | -1,0 | -1,3 | 1,2 | 1,8 | 2,3 | 1,2 | -1,3 | -1,1 | -1,7 | -1,4 | 0,8 | ± | 1,5 | -1,4 | ± | 0,2 | 0,61 |
| A_23_P122041 | PPP2CA | 1,1 | 1,2 | -1,8 | -2,6 | -2,1 | 1,1 | 1,0 | 1,1 | 1,2 | 1,0 | -0,5 | ± | 1,8 | 1,1 | ± | 0,1 | **0,01** |
| A_24_P107291 | PPP2R1B | 1,0 | 1,3 | -1,6 | -2,3 | -2,2 | 1,2 | -1,1 | -1,0 | -1,1 | -1,1 | -0,8 | ± | 1,6 | -0,5 | ± | 1,2 | 0,26 |
| A_24_P98762 | PPP2R1B | 1,5 | -1,1 | -7,1 | -1,9 | -1,3 | 1,4 | 1,7 | 1,5 | 1,2 | -1,3 | -1,0 | ± | 3,4 | 0,2 | ± | 1,5 | 0,61 |
| A_24_P388433 | PPP2R3A | 1,3 | -1,3 | -2,3 | -2,7 | 1,1 | 1,1 | -1,1 | 1,1 | 1,1 | 1,2 | -0,1 | ± | 1,9 | 0,0 | ± | 1,4 | 0,61 |
| A_23_P60458 | PPP2R4 | -1,2 | -1,6 | -2,7 | -3,6 | -1,2 | 1,4 | -1,6 | -1,4 | -2,5 | -2,0 | -1,4 | ± | 1,7 | -1,9 | ± | 0,4 | 0,91 |
| A_24_P369694 | PPP2R5C | -1,0 | 1,1 | -2,1 | -2,8 | -2,8 | -1,0 | 1,2 | 1,2 | 1,2 | 1,1 | -1,4 | ± | 1,5 | 1,1 | ± | 0,1 | 0,48 |
| A_24_P916496 | PRKCA | -2,2 | 1,9 | -1,1 | 1,2 | -4,0 | 1,1 | 5,9 | 5,4 | 2,4 | 1,9 | 0,1 | ± | 3,3 | 3,0 | ± | 1,9 | 0,07 |
| A_23_P205567 | PRKCH | -1,2 | 1,0 | -1,4 | -2,0 | -2,6 | -1,8 | -1,3 | 1,1 | 1,5 | -1,1 | -1,3 | ± | 1,3 | 0,0 | ± | 1,4 | 0,07 |
| A_33_P3294578 | PRKCI | 1,2 | 1,6 | -1,4 | -2,0 | -2,1 | -1,7 | -1,1 | 1,3 | 1,5 | 1,2 | -0,8 | ± | 1,6 | 0,8 | ± | 1,3 | 0,11 |
| A_23_P51187 | PRKCZ | 1,1 | -1,2 | 1,6 | 1,5 | 2,1 | -1,3 | -1,2 | 1,4 | 1,1 | -1,4 | 1,1 | ± | 1,2 | -0,7 | ± | 1,2 | 0,07 |
| A_23_P106016 | PRKD1 | 1,1 | 2,1 | -1,7 | -1,7 | -4,0 | 1,2 | 1,1 | 1,1 | 1,3 | 1,4 | -0,7 | ± | 2,1 | 1,5 | ± | 0,4 | 0,07 |
| A_23_P215406 | RAC1 | -1,0 | 1,3 | -1,7 | -2,7 | -2,6 | 1,2 | -1,2 | 1,0 | -1,0 | 1,0 | -1,0 | ± | 1,7 | 0,0 | ± | 1,3 | **0,04** |
| A_24_P228717 | RAC2 | -1,1 | -1,5 | -2,1 | -3,6 | -1,8 | 1,1 | -1,3 | -1,4 | -2,3 | -1,9 | -1,5 | ± | 1,5 | -1,7 | ± | 0,5 | 0,35 |
| A_33_P3295523 | RAC3 | -1,0 | -1,4 | -1,0 | 1,7 | 2,0 | -1,3 | -1,1 | 1,0 | -1,6 | -1,4 | 0,2 | ± | 1,5 | -1,4 | ± | 0,2 | 0,76 |
| A_24_P192262 | RALA | 1,3 | 1,2 | -1,4 | -2,9 | -2,4 | -1,2 | 1,1 | -1,0 | 1,2 | 1,2 | -1,3 | ± | 1,5 | 1,2 | ± | 0,1 | **0,02** |
| A_24_P497226 | RPS6KB1 | 1,1 | 1,4 | -1,6 | -2,4 | -3,0 | -1,2 | 1,2 | 1,2 | 1,6 | 1,5 | -1,0 | ± | 1,7 | 1,4 | ± | 0,2 | 0,11 |
| A_33_P3251727 | RYR2 | 1,1 | 1,4 | -5,2 | 1,2 | -1,0 | 1,0 | 1,0 | 1,1 | 1,3 | 2,2 | -0,3 | ± | 2,5 | 1,5 | ± | 0,5 | **0,01** |
| A_33_P3318606 | SHC2 | 2,1 | -1,1 | 2,3 | 1,1 | 1,7 | 2,9 | 1,0 | -1,8 | -1,6 | 1,1 | 1,4 | ± | 1,7 | -0,1 | ± | 1,4 | 0,07 |
| A_23_P343808 | SOS1 | -1,1 | -1,8 | -1,0 | 1,6 | 2,7 | 1,4 | -1,4 | -1,2 | -1,6 | -2,5 | 0,4 | ± | 1,7 | -1,8 | ± | 0,5 | 0,11 |
| A_33_P3250671 | TCF7 | 1,2 | -1,9 | 3,1 | 2,6 | 2,6 | -1,6 | -1,2 | -1,2 | -1,6 | -1,5 | 1,1 | ± | 2,1 | -1,5 | ± | 0,3 | **0,01** |
| A_23_P389588 | TCF7L2 | 1,7 | -1,5 | 3,3 | 7,4 | 4,4 | 1,4 | -1,2 | -1,4 | -1,1 | -2,6 | 2,8 | ± | 3,0 | -1,6 | ± | 0,7 | 0,07 |
| A_33_P3377294 | TCF7L2 | 1,0 | -1,6 | 1,1 | 1,5 | 2,2 | 1,3 | -1,6 | -1,4 | -2,5 | -1,8 | 1,0 | ± | 1,2 | -1,9 | ± | 0,4 | **0,04** |
| A_23_P411157 | WNT1 | -1,1 | 1,3 | -1,6 | -2,3 | -2,3 | -1,2 | 1,5 | -1,0 | -1,1 | 1,3 | -1,6 | ± | 0,6 | 0,7 | ± | 1,2 | **0,01** |
| A_33_P3382588 | WNT1 | 4,2 | 1,8 | 1,1 | 3,9 | -2,0 | 1,1 | -1,2 | 1,6 | 1,2 | 1,8 | 1,7 | ± | 2,3 | 0,9 | ± | 1,4 | **0,02** |
| A_23_P162322 | WNT10B | -1,2 | -1,2 | 2,5 | 5,4 | 2,3 | -1,2 | 1,8 | -1,2 | 1,6 | 1,1 | 1,1 | ± | 2,8 | 0,8 | ± | 1,4 | 1,00 |
| A_24_P253003 | WNT11 | -2,3 | -1,0 | -3,0 | -2,4 | -1,7 | -2,1 | -1,5 | 1,3 | -2,3 | 1,1 | -1,7 | ± | 1,5 | -0,9 | ± | 1,5 | 0,76 |
| A_23_P134601 | WNT16 | -1,0 | -1,1 | -9,6 | 1,3 | 1,1 | 1,1 | 1,2 | 1,0 | -1,1 | 1,0 | -1,0 | ± | 4,3 | 0,0 | ± | 1,2 | 0,26 |
| A_23_P138352 | WNT2B | 2,3 | -6,0 | 34,3 | 17,4 | 10,1 | -1,2 | -6,6 | -1,9 | -2,0 | -49,2 | 10,2 | ± | 13,9 | -16,0 | ± | 22,3 | 0,61 |
| A_23_P385690 | WNT3A | -1,1 | -1,0 | -1,1 | -1,3 | -2,4 | -4,0 | 1,6 | -1,3 | 2,8 | 1,2 | -1,9 | ± | 1,1 | 1,2 | ± | 1,6 | **0,01** |
| A_33_P3417502 | WNT3A | -1,1 | -1,7 | 1,0 | 1,2 | 2,3 | 1,2 | -1,6 | -1,5 | -2,4 | -1,7 | 0,5 | ± | 1,5 | -1,8 | ± | 0,4 | **0,01** |
| A_23_P258410 | WNT7A | 1,3 | 1,0 | 1,3 | -2,9 | -2,2 | -1,4 | 1,1 | -1,2 | -1,1 | -2,2 | -0,9 | ± | 1,8 | -0,3 | ± | 1,6 | **0,01** |
| A_33_P3248903 | WNT7B | -1,0 | 1,5 | 3,4 | 1,9 | 2,2 | 1,5 | 1,5 | -1,4 | 1,3 | 1,3 | 1,1 | ± | 1,9 | 1,4 | ± | 0,1 | 0,76 |
| A_24_P911607 | WNT7B | 1,1 | -3,2 | -2,7 | -2,2 | -10,1 | -1,1 | -2,6 | -1,1 | -1,0 | 1,7 | -2,7 | ± | 3,9 | -1,3 | ± | 2,2 | 0,61 |
| A_33_P3256920 | WNT7B | 1,4 | -1,5 | -2,1 | -1,5 | 1,4 | 1,4 | 1,3 | -2,2 | 1,2 | -1,8 | -0,3 | ± | 1,8 | -0,2 | ± | 1,7 | 0,76 |
| A_24_P314351 | ZBTB33 | 1,0 | 1,5 | -1,5 | -2,6 | -2,5 | -1,0 | 1,1 | 1,1 | 1,4 | 1,3 | -0,9 | ± | 1,6 | 1,3 | ± | 0,2 | 0,76 |

Supplementary Table 4: Statistical overview of clinical outcome and gene expression of the candidate genes in n=50 LCLs. Gene expression was compared between positive (e.g. responder) and negative (e.g. non-responder) clinical outcome by the use of t-test (basal gene expression) or Wilcoxon rank sum test (fold changes). Significant p-values are bold and underlined.

|  |  | **WNT2B** | **SULT4A1** | **TCF7L2** | **FZD7** | **ABCB1** |
| --- | --- | --- | --- | --- | --- | --- |
| basal gene expression | Response after 5 weeks | 0.795 | **0.029** | 0.595 | 0.422 | 0.979 |
|  | Response after 8 weeks | 0.877 | 0.092 | 0.430 | 0.476 | 0.635 |
|  | Remission after 5 weeks | 0.324 | 0.604 | 0.490 | 0.925 | 0.472 |
|  | Remission after 8 weeks | 0.656 | 0.354 | 0.109 | 0.187 | 0.992 |
| fold changes after treatment with fluoxetine | Response after 5 weeks | 0.819 | 1.000 | 0.438 | 0.272 | 0.535 |
|  | Response after 8 weeks | 0.966 | 1.000 | 0.373 | 0.235 | 0.963 |
|  | Remission after 5 weeks | **0.025** | 0.229 | 0.159 | 0.357 | 0.266 |
|  | Remission after 8 weeks | 0.316 | 0.400 | 0.224 | 0.462 | 0.760 |
